# The Roles of Floral Organ Genes in Regulating Rosaceae Fruit Development

**DOI:** 10.3389/fpls.2021.644424

**Published:** 2022-01-05

**Authors:** Jia-Long Yao, Chunying Kang, Chao Gu, Andrew Peter Gleave

**Affiliations:** ^1^The New Zealand Institute for Plant and Food Research Limited, Auckland, New Zealand; ^2^College of Horticulture and Forestry, Huazhong Agricultural University, Wuhan, China; ^3^State Key Laboratory of Crop Genetics and Germplasm Enhancement, Nanjing Agricultural University, Nanjing, China

**Keywords:** AP2, MADS-box, miR172, fruit development, apple, peach, strawberry

## Abstract

The function of floral organ identity genes, *APETALA1/2/3*, *PISTILLATA, AGAMOUS*, and *SEPALLATA1/2/3*, in flower development is highly conserved across angiosperms. Emerging evidence shows that these genes also play important roles in the development of the fruit that originates from floral organs following pollination and fertilization. However, their roles in fruit development may vary significantly between species depending on the floral organ types contributing to the fruit tissues. Fruits of the Rosaceae family develop from different floral organ types depending on the species, for example, peach fruit flesh develops from carpellary tissues, whereas apple and strawberry fruit flesh develop from extra-carpellary tissues, the hypanthium and receptacle, respectively. In this review, we summarize recent advances in understanding floral organ gene function in Rosaceae fruit development and analyze the similarities and diversities within this family as well as between Rosaceae and the model plant species Arabidopsis and tomato. We conclude by suggesting future research opportunities using genomics resources to rapidly dissect gene function in this family of perennial plants.

## Introduction

Floral organs are classified into four types, sepals, petals, stamens, and carpels. Their development is regulated by four different classes of floral organ identity genes according to the proposed ABCE model (Coen and Meyerowitz, 1991; Weigel and Meyerowitz, 1994; Krizek and Fletcher, 2005). In summary, for *Arabidopsis* sepal formation is specified by the class A genes *APETALA1* (*AP1*) and *AP2*, petal formation is specified by the class A genes and two class B genes [*APETALA3* (*AP3*) and *PISTILLATA* (*PI*)]; stamen formation requires *AP3* and *PI* together with the class C gene *AGAMOUS* (*AG*); and carpel formation is controlled by the *AG* alone (Weigel and Meyerowitz, 1994). Proper development of all four whorls of floral organs also requires the class E *SEPALLATA* (*SEP*) genes. The consequence of mutating all four *SEPALLATA* genes (*sep1, sep2, sep3, and sep4*) simultaneously is a plant that produces flowers consisting of reiterating whorls of leaf-like organs (Krizek and Fletcher, 2005). All class A, B, C, and E genes encode MADS-box transcription factors, except for *AP2*, which encodes a transcription factor belonging to the super-family of AP2/ERF proteins (Okamuro et al., 1997). However, classification of a gene as being a member of the ABCE model is based on its function and not sequence similarity to other members of the ABCE model.

The diversity of fruit types seen between various flowering plant species is primarily a consequence of different carpellary and/or extra-carpellary floral tissues contributing to fruit formation (Spjut, 1994). Botanical fruit, also known as true fruit, is derived from carpellary tissues, whereas accessory fruit, also known as pseudo fruit, has fruit flesh derived from extra-carpellary tissues (Liu et al., 2020). With fruit being derived from floral organs, the genes that regulate the growth of floral organs also play important roles in the control of fruit development. This was clearly demonstrated by the functions of the Arabidopsis *FRUITFULL* (*FUL*), an *AP1* homolog (Gu et al., 1998), tomato *TM29*, a *SEPALLATA* homolog (Ampomah-Dwamena et al., 2002), and poppy *AP1/FUL* homologs (Pabón-Mora et al., 2012).

Rosaceae species produce flowers with five sepals and petals, and a varied number of stamens and carpels. Their carpels show variations in shape, degrees of fusion, and relative position to other floral organs. These variations are the key contributing factors of fruit type diversity in Rosaceae (Liu et al., 2020). For example, peach develops a superior ovary as the single carpel is positioned above all other floral organs and goes on to develop a drupe. In contrast, apple has five fused carpels that are below the attachment point of all other floral organs, develops an inferior ovary that fuses with the surrounding hypanthium tissues formed from the bases of sepals, petals, and stamens and develops a pome (Liu et al., 2020).

In addition to floral structure variations, differential enlargement of specific floral tissues also contributes to fruit type diversity in Rosaceae. Strawberry and raspberry, for example, have a similar floral structure that consists of numerous carpels growing on a receptacle. Strawberry is an achenetum because its carpels are not enlarged after fertilization and become dry achenes, but the receptacle is enlarged and grows into the fleshy pseudo fruit. In contrast, raspberry is a drupetum because its carpels are enlarged after fertilization to form fleshy drupelets but the receptacle is not enlarged or fleshy (Liu et al., 2020).

Together with flower and fruit morphological differences, duplicated floral organ genes have the opportunity to play diversified roles in fruit development. In Rosaceae, whole-genome duplication (WGD) events have resulted in gene copies and their potential to have dosage effects, redundancy of function, or for copies to have undergone functional changes, such as neo-functionalization (one gene copy taking on a totally new function) or sub-functionalization (each gene copy retaining a subset of the original ancestral functions; Conant et al., 2014; Xiang et al., 2017). In the following sections, we summarize recent advances in understanding floral organ gene duplication, expression profiles, and function in relation to Rosaceae fruit development. We also analyze the similarities and diversities within this family predominantly using apple, peach, and strawberry as examples representing the diversity of fruit types in the family. The similarities and diversities between Rosaceae and other plants, in particular Arabidopsis and tomato, are also analyzed.

## Gene Duplication and Fruit Expression Pattern Diversity of Floral Organ Genes Indicate Their Diversified Functions in Regulating Fruit Development

Prior to the assembly of reference genome sequences, sequence-based approaches using homology to the *Arabidopsis* genes were used to clone and identify floral organ MADS-box genes from Rosaceae species. In apple, several studies identified a total of two *AP1-*related genes, *MdMADS5* (*MdAP1*; Yao et al., 1999; Kotoda et al., 2002) and *MdMADS2* (Sung et al., 1999), one *PI* homolog, *MdPI* (Yao et al., 2001), two *AP3* homologs, *MdMADS13* (Van Der Linden et al., 2002) and *MdTM6* (Kitahara et al., 2004), three *AG*-related genes, *MdMADS10* (Yao et al., 1999), *MdMADS14*, and *MdMADS15* (Van Der Linden et al., 2002), and five *SEP*-related genes, *MdMADS4* (Sung et al., 2000), *MdMADS6*, *7*, *8*, and *9* (Yao et al., 1999). After the first apple reference genome was assembled (Velasco et al., 2010), three additional *SEP-*related genes were identified, *MdMADS18*, *104*, and *118* (Ireland et al., 2013). All these genes, except for *MdPI*, have been annotated in the second apple reference genome generated using a haploid plant derived from the cultivar “Golden Delicious” (Daccord et al., 2017).

**Figure 1:**
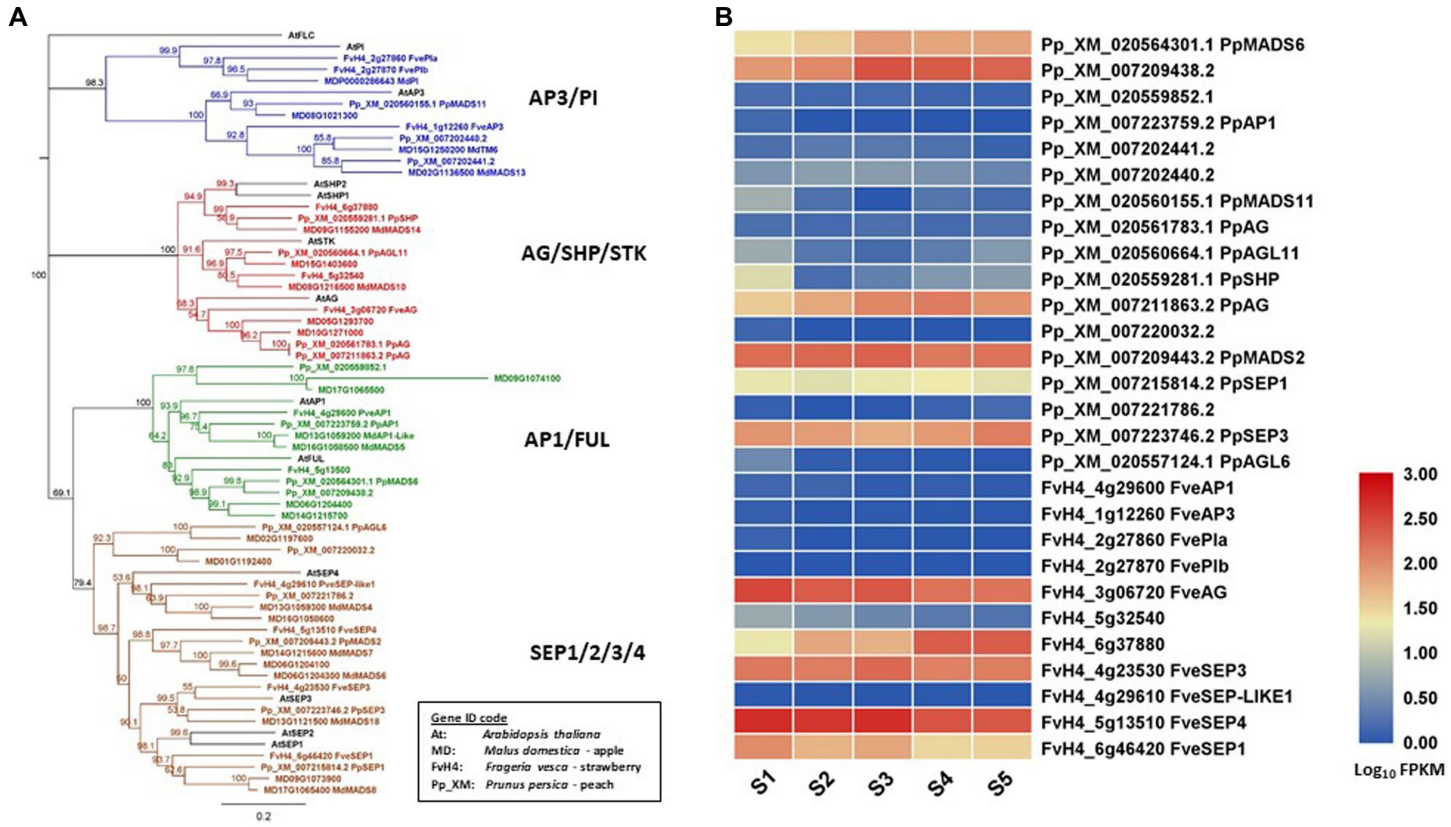
Phylogeny tree of floral MADS-box proteins and their gene expression in developing fruit tissues. **(A)** a Neighbor-Joining consensus tree was constructed using Geneious software (v10.0.9) with 1,000 bootstrap replicates after protein sequences were aligned using Geneious Alignment software (v10.0.9). MADS-box protein sequences downloaded from the reference genome of apple (Daccord et al., 2017), peach (Verde et al., 2013), strawberry (Edger et al., 2017), and Arabidopsis. The Arabidopsis protein IDs are AT1G69120.1 (AtAP1), AT5G60910.1 (AtFUL), AT5G20240.1(AtPI), AT3G54340.1 (AtAP3), AT4G18960.1 (AtAG), AT3G58780.1 (AtSHP1), AT2G42830.1 (AtSHP2), AT4G09960.1 (AtSTK), AT5G15800.1 (AtSEP1), AT3G02310.1 (AtSEP2), AT1G24260.1 (AtSEP3), AT2G03710.1 (AtSEP4), and AT5G10140.1 (AtFLC). **(B)** a heatmap shows the expression patterns of floral MADS-box genes in fruit tissues of peach and strawberry at five developmental stages (S1-S5) based on previously published RNA-seq data (Pei et al., 2020). Rosaceae Gene IDs are coded as: MD for apple (*Malus domestica*), Pp_XM for peach (based on Prunus_persica_NCBIv2) and FvH4 for strawberry (based on *Frageria vesca* H4). S1 to S5: 21, 42, 56, 84, and 105 days after full bloom for peach, and 11, 27, 31, 43, and 47 DAFB for strawberry.

In this review, floral MADS-box genes are identified from the reference genome of peach (Verde et al., 2013), strawberry (Edger et al., 2017), and apple (Daccord et al., 2017). Their gene IDs are listed in Table 1 together with their class, based on gene lineage, and the relevant references. Their relationship is shown in a protein sequence-based phenology tree in Figure 1A. One or more genes from peach, strawberry, and apple are identified as homologs of each of the Arabidopsis floral MADS-box genes. In apple, 12 floral MADS-box genes are present as homolog pairs, most likely due to a recent apple-specific whole-genome duplication (Velasco et al., 2010).

**Table 1:** Floral MADS-box and AP2 genes in peach, strawberry, and apple.

Species	Gene ID	Class	Gene name	References
**Floral MADS-box**				
Peach	Pp_XM_020564301.1	FUL	*PpMADS6*	Xu et al., 2014
	Pp_XM_007209438.2	FUL		
	Pp_XM_020559852.1	AP1		
	Pp_XM_007223759.2	AP1	*PpAP1*	Zhang et al., 2008
	Pp_XM_007202441.2	AP3		
	Pp_XM_007202440.2	AP3		
	Pp_XM_020560155.1	AP3	*PpMADS11*	
	Pp_XM_020561783.1	AG	*PpAG* 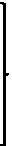	Tani et al., 2009
	Pp_XM_020560664.1	STK	*PpAGL11*	
	Pp_XM_020559281.1	SHP	*PpSHP*	
	Pp_XM_007211863.2	AG	*PpAG*	
	Pp__007220032.2	SEP		
	Pp_XM_007209443.2	SEP	*PpMADS2* 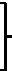	Xu et al., 2008
	Pp_XM_007215814.2	SEP	*PpSEP1*	
	Pp_XM_007221786.2	SEP		
	Pp_XM_007223746.2	SEP	*PpSEP3* 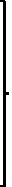	Xu et al., 2008
	Pp_XM_020557124.1	SEP	*PpAGL6*	
Strawberry	FvH4_4g29600	AP1	*FveAP1* 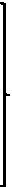	Hollender et al., 2014
	FvH4_1g12260	AP3	*FveAP3*	
	FvH4_2g27860	PI	*FvePIa*	
	FvH4_2g27870	PI	*FvePIb*	
	FvH4_3g06720	AG	*FveAG*	
	FvH4_5g13500	FUL		
	FvH4_5g32540	STK		
	FvH4_6g37880	SHP		
	FvH4_4g23530	SEP	*FveSEP3* 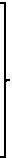	Hollender et al., 2014
	FvH4_4g29610	SEP	*FveSEP-LIKE1*	
	FvH4_5g13510	SEP	*FveSEP4*	
	FvH4_6g46420	SEP	*FveSEP1*	
Apple	MD06G1204400	FUL		
	MD13G1059200	AP1	*MdAP1-like*	Van Der Linden et al., 2002
	MD14G1215700	FUL		
	MD17G1065500	AP1		
	MD09G1074100	AP1		
	MD16G1058500	AP1	*MdMADS5*	Yao et al., 1999; Kotoda et al., 2002
	MD02G1136500	AP3	*MdMADS13*	Van Der Linden et al., 2002
	MD15G1250200	AP3	*MdTM6*	Kitahara et al., 2004
	MD08G1021300	AP3		
	MDP0000286643	PI	*MdPI*	Yao et al., 2001, 2018
	MD05G1293700	AG	*MdAMDS22*	Klocko et al., 2016
	MD08G1216500	STK	*MdMADS10*	Yao et al., 1999
	MD09G1155200	SHP	*MdMADS14*	Van Der Linden et al., 2002
	MD10G1271000	AG	*MdMADS15*	Klocko et al., 2016
	MD15G1403600	STK		
	MD01G1192400	SEP		
	MD06G1204100	SEP		
	MD06G1204300	SEP	*MdMADS6*	Yao et al., 1999; Ireland et al., 2013
	MD09G1073900	SEP		
	MD13G1059300	SEP	*MdMADS4*	Sung et al., 2000
	MD13G1121500	SEP	*MdMADS18*	Ireland et al., 2013
	MD14G1215600	SEP	*MdMADS7*	Yao et al., 1999; Ireland et al., 2013
	MD16G1058600	SEP		
	MD17G1065400	SEP	*MdMADS8*	Yao et al., 1999; Ireland et al., 2013
	MD02G1197600	SEP		
**Floral AP2**				
Peach	XM_007205081.2	AP2	*PpTOE1a* 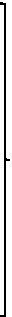	Gattolin et al., 2018
	XM_007220804.2	AP2	*PpTOE2*	
	XM_007207942.2	AP2	*PpAP2*	
	XM_020566630.1	AP2		
	XM_020565001.1	AP2	*PpTOE1b*	
Strawberry	FvH4_1g16350	AP2	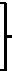	Hollender et al., 2014
	FvH4_7g20380	AP2		
	FvH4_3g33940	AP2		
	FvH4_6g15180	AP2		
Apple	MD01G1113400	AP2	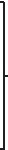	Yao et al., 2015
	MD02G117600	AP2		
	MD03G1107900	AP2		
	MD04G1105200	AP2		
	MD07G1180900	AP2		
	MD11G1121200	AP2		
	MD12G1125900	AP2		
	MD15G1286400	AP2		

Expression patterns of *SEP*-*like* genes in apple fruit have been described previously, such as the *AtSEP1/2*-like *MADS1/MADS8*, and *MADS9* showing high expression at two to seven days after pollination, the *PhFBP9/23*-like *MADS3/MADS7* and *MADS6* showing high expression at four to eight weeks after pollination, and the *AtSEP4*-like *MADS4* showing strong expression in young fruits (Sung and An, 1997; Yao et al., 1999; Sung et al., 2000). In addition, Yao et al. (1999) demonstrated the expression of class *AP1-* and *AG*-related genes in the fruit flesh of apple. Detection of their expression in fruit indicates that *AP1*-, *AG*-, and *SEP*-related *MADS*-box genes have a potential role in apple fruit development.

Transcriptome analyses of whole fruit tissues without seeds collected from five developmental stages revealed that two *FUL* homologs (*PpMADS6* and XM_007209438), an *AG* homolog (*PpAG*), and three *SEP* homologs (*PpMADS2, PpSEP1*, and *PpSEP3*) are expressed in peach fruit. The same analyses showed that an *AG* homolog (*FveAG*), a *SHP* homolog (FvH6g37880), and three *SEP* homologs (*FveSEP1/3/4*) are expressed in strawberry (*Fragaria* x *ananassa*) fruit (Pei et al., 2020; Figure 1B). In addition, two *AP3*-related genes (XM007202440 and *PpMADS11*) are also expressed in peach fruit during development although their expression levels are relatively low (Figure 1B). The expression of *AP3*-related genes in peach fruit is interesting because this expression is not detected in apple or strawberry fruit. In contrast, expression of *AP1/FUL* homologs is at a high level in apple and peach but not detected in strawberry. It would be worthwhile determining the functions of the*AP1/FUL* and *AP3/PI* homologs in regulating fruit development of Rosaceae.

**Figure 2:**
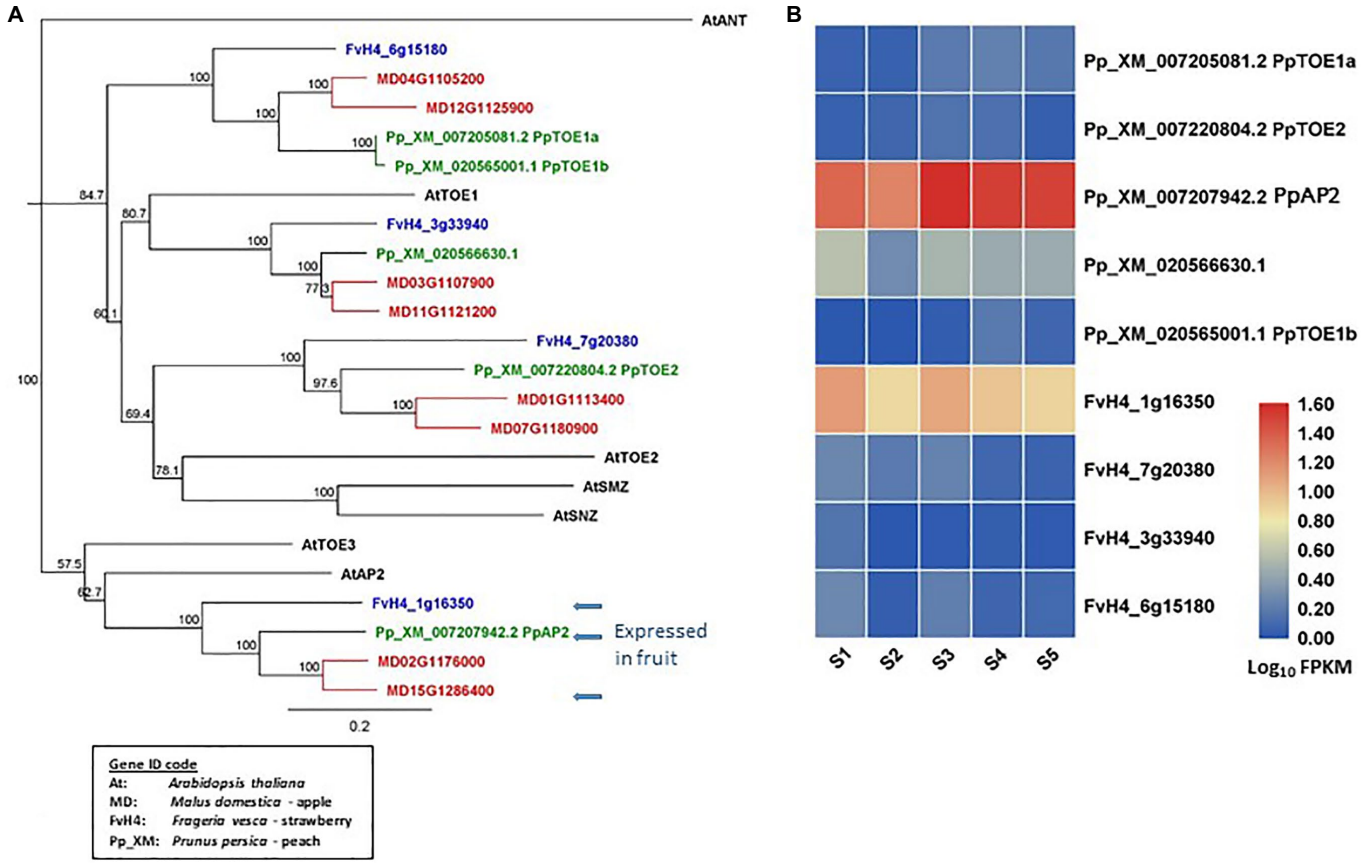
Phylogeny tree of AP2-like proteins and their gene expression in developing fruit tissues. **(A)** a Neighbor-Joining consensus tree was constructed using Geneious software (v10.0.9) with 1,000 bootstrap replicates after protein sequences were aligned using Geneious Alignment software (v10.0.9). AP2-like protein sequences downloaded from the reference genome of apple, peach, strawberry, and Arabidopsis. The Arabidopsis protein IDs are AT4G36920.1 (AtAP2), AT2G28550.3 (AtTOE1), AT5G60120.2 (AtTOE1), AT5G67180.1 (AtTOE3), AT3G54990.1 (AtSMZ), AT2G39250.1 (AtSNZ), and AT4G37750.1 (AtANT). **(B)** a heatmap shows the expression patterns of *AP2-like* genes in fruit tissues of peach and strawberry at five developmental stages (S1-S5) based on previously published RNA-seq data (Pei et al., 2020). Rosaceae Gene IDs are coded as: MDs for apple (*Malus domestica*), Pp_XM for peach (based on Prunus_persica_NCBIv2), and FvH4 for strawberry (based on *Frageria vesca* H4). S1 to S5: 21, 42, 56, 84, and 105 days after full bloom for peach, and 11, 27, 31, 43, and 47 DAFB for strawberry. The arrows indicate *AP2* genes predominantly expressed in fruits.

AP2 homologous genes have been identified from the reference genomes of apple (Daccord et al., 2017), peach (Verde et al., 2013), and strawberry (Edger et al., 2017; Figure 2A). In apple, there are eight *euAP2* (Kim et al., 2006) homologs that contain a miR172 target site in the coding sequences and encode proteins containing two AP2 domains (Yao et al., 2015). In peach and strawberry, five and four such AP2 homologs have been identified, respectively. The AP2 homologs of these species are classified into four distinct clades. Each clade contains two apple and one strawberry homolog. One clade contains two peach homologs and of other three clades each contains one peach homolog (Figure 2A). The specific duplication of AP2 homologs in apple provides new potential of dosage effect or functional changes. Among the eu*AP2* homologs, one in each species (MD15G1286400, FvH_1g16350, and Pp_XM_007207942) is expressed predominantly in the developing fruit tissues (Yao et al., 2015; Pei et al., 2020; Figures 2A,B). These three homologs are in the same clade as Arabidopsis AP2 (AtAP2) in the phylogenetic tree (Figure 2A). Further insights into the roles of these floral MADS-box and eu*AP2-like* genes in fruit development may be obtained by analyzing their expression patterns using transcriptome data sets generated in Rosaceae species (Kang et al., 2013; Hawkins et al., 2017; Hu et al., 2018; Pei et al., 2019; Shahan et al., 2019; Tan et al., 2019; Ying et al., 2019; Shang et al., 2020).

From the above analyses of gene sequences and expression patterns, it is clear that these three species in the Rosaceae family contain similar genes in the floral MADS-box and AP2 clades, but the gene number varies among the three species (Figure 2A). Expression of these genes generally shows a degree of similarity across the three species described, although specific temporal and spatial expression patterns are present (Figure 2B; Kang et al., 2013; Hawkins et al., 2017; Hu et al., 2018; Pei et al., 2019; Shahan et al., 2019; Tan et al., 2019; Ying et al., 2019; Shang et al., 2020). Evidence to date would indicate that there are conserved and diversified functions for these genes in different species, although the research to understanding these functions is very much in its infancy.

## Mir172 and Ap2 Have Diversified Roles in Regulating Fruit Development

In plants, microRNA172 (miR172) is highly conserved and targets a subfamily of *AP2-like* genes to repress their expression, by initiating degradation and/or inhibiting translation of the target mRNA (Aukerman and Sakai, 2003; Chen, 2004; Zhu et al., 2009; Zhu and Helliwell, 2011). As members of the euAP2 subfamily have different spatial and temporal patterns of expression (Okamuro et al., 1997; Tang et al., 2007; Yao et al., 2015) and interact with different proteins (Teotia and Tang, 2015), the *MIR172* gene family has the potential to affect a number of aspects of plant development.

In annual plant species, early flowering is a phenotypic change commonly observed for over-expression of miR172 (Aukerman and Sakai, 2003; Chen, 2004; Mlotshwa et al., 2006; Zhu and Helliwell, 2011; Teotia and Tang, 2015), and in some instances abnormalities of the floral organs have been observed (Mlotshwa et al., 2006; Zhu et al., 2009). As AP2 governs floral organ size (Jofuku et al., 2005) and floral organ development (Yant et al., 2010), then miR172 has the potential to influence growth of fruit.

In *Arabidopsis*, miR172 positively regulates *Arabidopsis* silique growth (Ripoll et al., 2015). The silique is derived from carpel tissues and its growth is negatively regulated by AP2 (Ripoll et al., 2011). The expression of AP2 is repressed in the silique valve by miR172 (Ripoll et al., 2015). Therefore, suppression of the function of miR172, through the use of microRNA mimicry technology, results in a reduction in silique size. Similarly, a reduction in the size of the silique occurs in transgenic plants expressing a modified *AP2* gene without a functional miR172-target sequence (Ripoll et al., 2015).

**Figure 3:**
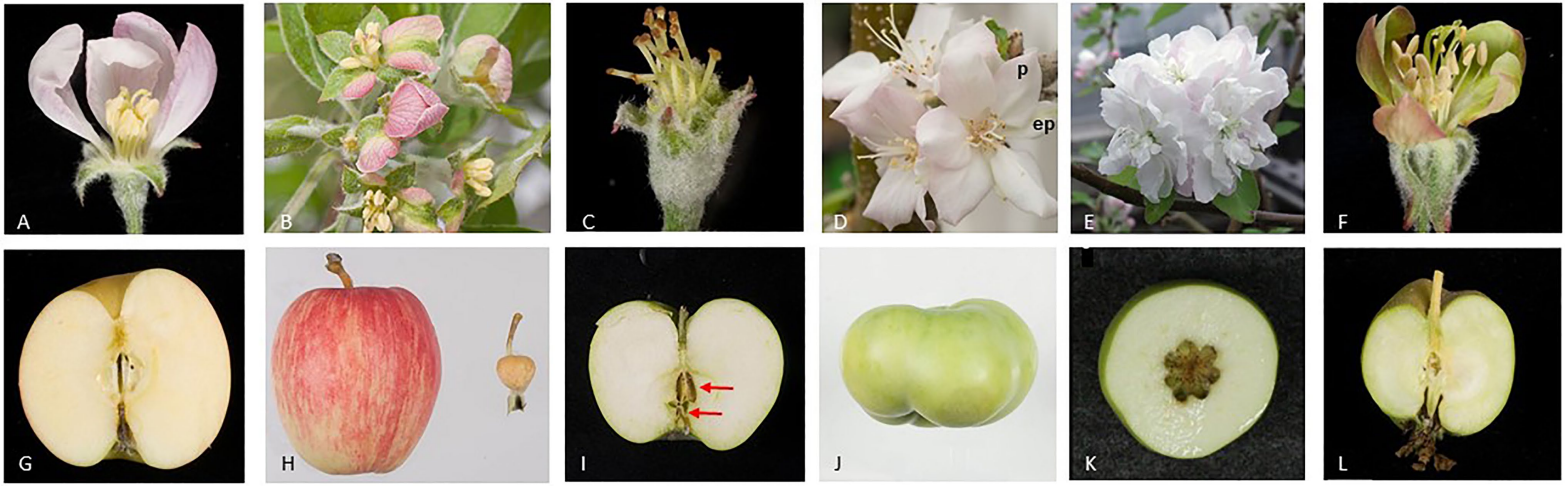
Apple flower and fruit phenotypes after alteration of the expression of floral organ genes. The images show open flowers **(A–F)** and mature fruit **(G–L)** of wild-type **(A,G)** and transgenic apple plants **(B–F, H–L)**. miR172 is known to inhibit the expression of the *AP2-* and *AP2-like* genes. miR172 over-expression converts sepal segments to petal-like tissues **(B)** outer petals are removed to show sepal-petal conversion and dramatically reduces fruit size **(H)**, wild-type fruit on left and transgenic fruit on right (Yao et al., 2015). Repressing expression of the *MdPI* gene converts petals to sepals, and stamens to carpels **(C)**, and results in parthenocarpic (seedless) fruit with two whorls of carpels marked using two red arrows **(I)**. However, over-expressing *MdPI* converts sepals to petals (p, petal; ep, ectopic petal) **(D)** and suppresses fruit tissue growth resulting in altered fruit shape **(J)** (Yao et al., 2001, 2018). Repressing expression of two *AG* homologs, *MdMADS15/22*, converts stamens to petals **(E)** and increases carpel number and therefore fruit locule number **(K)** (Klocko et al., 2016). Repressing expression of two *SEP* homologs, *MdMADS8/9*, partially converts petals to greenish sepal-like organs **(F)** and suppresses fruit flesh growth **(L)** (Ireland et al., 2013).

Apple fruit flesh, is derived largely from the hypanthium, hypothesized to consist of the fused bases of the stamens, petals, and sepals (Pratt, 1988) and fruit core is derived from the carpellary tissues. Further molecular studies have indicated that sepal bases make the greatest contribution to apple flesh formation (Yao et al., 2001). Negative regulation of apple fruit growth by miR172 was demonstrated in transgenic plants over-expressing miR172 and also observed in natural variants of apple. Over-expression of miR172 in apple converted segments of sepals to petal-like tissues, indicating that AP2-like transcription factors are required for sepal development and furthermore the miR172 over-expression reduced fruit weight more than 60-fold (Figure 3; Yao et al., 2015). A transposable element (TE) insertional allele of the apple *MIR172p* gene is associated with reduction of *MIR172* expression and co-located with one of four fruit size QTLs (Yao et al., 2015). In the progeny of a controlled cross, the TE insertional allele is associated with large fruit and underlies the evolution of fruit size from the small wild crabapples to large cultivated apples. Although the correlation between fruit size and the level of AP2 expression during fruit development could be interpreted as a direct effect of AP2 on fruit growth, one cannot discount that this is an indirect effect because AP2 represses the function of class C genes (e.g., *AG*) in the first whorl, according to the ABCE model (Wollmann et al., 2010). Therefore, miRNA-mediated reduction of AP2, resulting in elevated expression of class C genes in the first whorl during flower development, could lead to a conversion of sepals to carpel-like structures. As sepals contribute to the apple fruit flesh we postulate that this conversion of floral organs could account for the reduction in fruit size. However, a sepal to carpel conversion in apple over-expressing miR172 was not reported (Yao et al., 2015).

In tomato, over-expression of miR172 produced flowers consisting entirely of ovary tissues that develop into parthenocarpic fruit, with a fruit-in-fruit phenotype observed in some extreme cases (Yao et al., 2016). As in apple, the size of the tomato fruit was reduced significantly compared with wild-type control fruit, although the tomato fruit size reduction (4-5-fold) was not as dramatic as that observed in apple. Growth of tomato fruit flesh tissue is dependent largely on division and expansion of cells occurring post-fertilization through the stimulation by hormones released from the seeds. Although over-expression of miR172 may promote fruit development in the absence of fertilization, the parthenocarpic fruit is smaller than wild-type fruit. This “smaller than wild-type” size of plants over-expressing miR172 indicates that the direct or indirect influence of the miR172 transgene on promoting tomato fruit growth in these parthenocarpic fruit does not fully compensate for the growth stimulation of seed-derived hormones in wild-type fruit.

**Figure 4:**
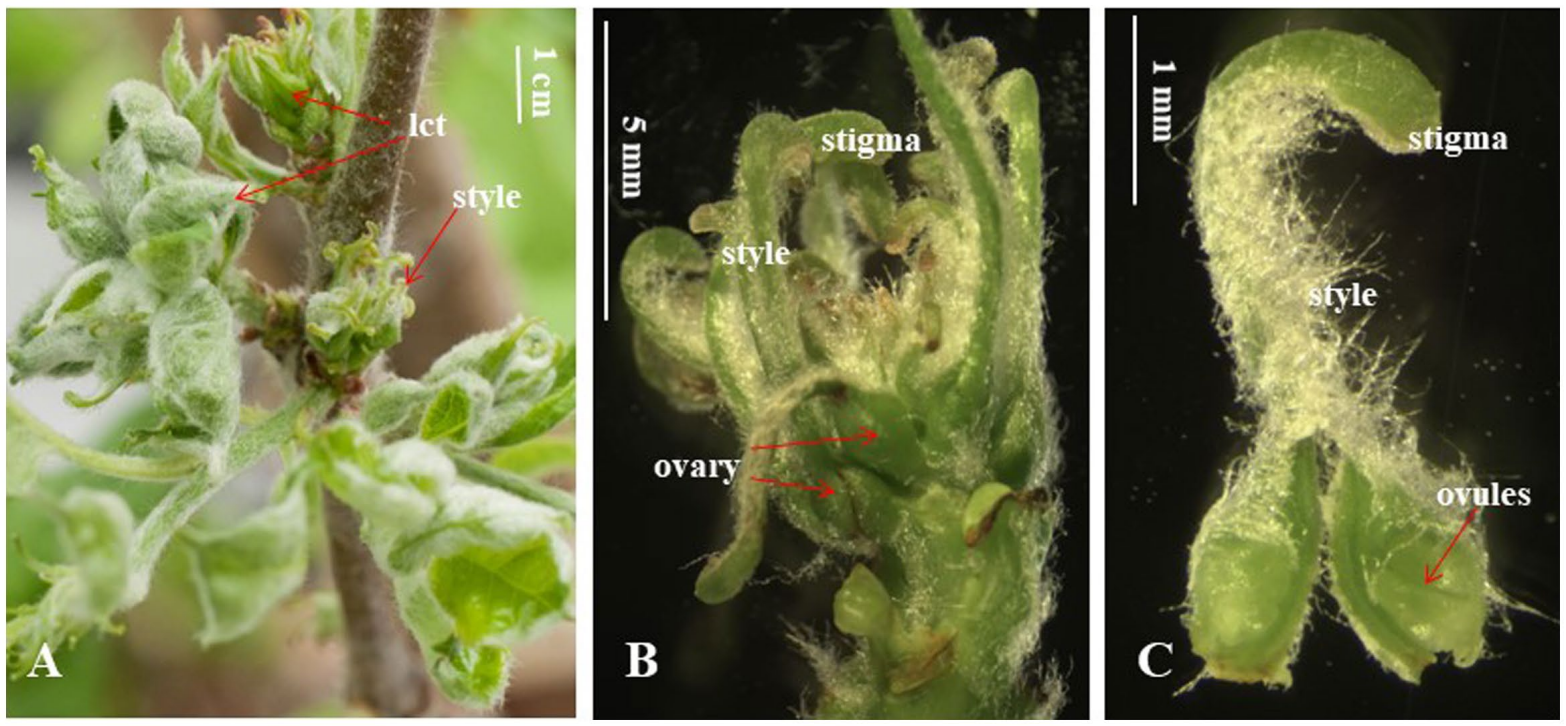
Flowers of apple transgenic plants over-expressing miR172. **(A)** Floral inflorescences show abnormal flowers consisting of leaf-like carpel tissues (lct), style, and stigma, but no sepals, petals, or stamens. **(B)** A florescence of **(A)** with leaf-like carpels removed reveals separate carpels consisting of ovary, style, and stigma. **(C)** A single carpel removed from the inflorescence in **(B)** reveals two ovules after cutting the ovary open (This figure is re-produced from Yao et al., 2015).

The reports described above have shown miR172/AP2 playing different roles in the regulation of the development of different fruit types represented by apple, Arabidopsis, and tomato. This suggests that miR172/AP2 has the potential for a diversified function in regulating the development of different fruits of the Rosaceae family, although to date, their function has not been reported in any Rosaceae species other than apple. It is interesting to note that the extreme phenotype of apple transgenic plants over-expressing miR172 displays abnormal flowers without sepals, petals, or stamens, but with separate carpels rather than the fused the carpels of normal apples, and no hypanthium surrounding the ovary (Figure 4; Yao et al., 2015). If these flowers were to develop into fruit, the fruit would resemble peach rather than apple, indicating miR172/AP2 could be involved in the diversification of fruit types in Rosaceae.

## *Pi/Ap3* Homologs Negatively Regulate Fruit Growth

The Arabidopsis *PI* and *AP3* are single-copy class B genes, expressed specifically in petals and stamens during their development (Goto and Meyerowitz, 1994; Jack et al., 1992). In *PI* or *AP3* loss of function mutants, the flower phenotype is double the number of carpels, two whorls of sepals, and the absence of stamens or petals (Goto and Meyerowitz, 1994; Jack et al., 1992). Three apple varieties, Rae Ime, Spencer Seedless, and Wellington Bloomless, produce abnormal flowers that resemble Arabidopsis *pi* and *ap3* mutants. Interestingly, the flowers of these three apple varieties develop parthenocarpic fruit, and their single-copy *MdPI* gene is not expressed owing to the presence of a long-terminal-repeat TE (Yao et al., 2001). Consistently, a similar flower phenotype and parthenocarpic fruit development are achieved with *MdPI* cosuppression in apple transgenic plants (Yao et al., 2018). The parthenocarpic trait of apple in *MdPI* loss of function mutants and cosuppression transgenic plants may be explained by the conversion of petals to sepals that are the key floral organs contributing to apple flesh tissue growth (Yao et al., 2001). An additional or alternative explanation for this parthenocarpic trait is that the loss of stamens, as a consequence of suppression of the class B *MdPI*, results in male sterility. Male sterility has been associated with parthenocarpic fruit production in multiple plant species, including tomato (Ingrosso et al., 2011; Medina et al., 2013; Takei et al., 2019), citrus (Vardi et al., 2008), and grape (Royo et al., 2016). In contrast, ectopic *MdPI* expression coverts sepals to petals and inhibits apple fruit growth toward the base of the fruit resulting in short and flat fruit (Figure 3). Histological analyses showed that suppression of cell expansion was likely to be the key reason for the reduced tissue growth, although the possibility of inhibition of cell division could not be ruled out (Yao et al., 2018). These two studies clearly indicated *MdPI* plays an inhibitory role on fruit growth.

The inhibitory role of the class B genes in fruit growth is also supported by studies in other plant species. For example, ectopic expression of *VvPI*, caused by an insertion of a miniature inverted-repeat TE, inhibits the growth of fruit flesh tissue and results in small, fleshless berries in grape (*Vitis vinifera*; Fernandez et al., 2013). In tomato, *DEFICIENS* (an *AP3* homolog) expression is temporarily upregulated before and throughout anthesis in the wild-type but not in the *parthenocarpic fruit* (*pat*) mutant (Mazzucato et al., 2008), and silencing of *TM6* (an *AP3* homolog) expression using RNA interference produced parthenocarpic fruit (De Martino et al., 2006). These results suggest that the *AP3* and *PI* homologs in other fruit crops also may suppress ovary and fruit development.

In Arabidopsis, PI and AP3 function together through forming protein heterodimers in petals and stamens (Goto and Meyerowitz, 1994). As apple and grape have several copies of *AP3* paralogues, some have acquired new expression patterns, like the sepal and fruit tissue expression observed in apple (Van Der Linden et al., 2002; Kitahara et al., 2004) and grape (Poupin et al., 2007). Ectopic expression of *VvPI* in grape or *MdPI* in apple inhibits fruit flesh tissue growth (Fernandez et al., 2013; Yao et al., 2018). A possible explanation for this phenotype in apple and grape is that the ectopic expression of PI and the new expression patterns of AP3 homologues results in the concomitant presence of AP3 and PI in fruit flesh tissue allowing the formation of functional AP3/PI heterodimers which repress fruit flesh tissue growth.

In cultivated strawberry (*Fragaria* x *ananassa*), two *AP3* homologues (*FaAP3* and *FaTM6*) exhibited an expression pattern equivalent to that of *AP3* in Arabidopsis. CRISPR/Cas9-mediated genome editing of strawberry to knockout *FaTM6* inhibited anther development although normal fruit development occurred after pollination with wild-type pollen (Martin-Pizarro et al., 2019), indicating *FaTM6* plays an important role in strawberry anther development. Comparison of the phenotypes between *MdPI* mutants of apple and *FaTM6* knockout of strawberry indicates the function of AP3/PI homologs is conserved in regulating floral organ development, but different in promoting fruit growth.

## *Ag/Shp*-Related Genes Regulate Apple And Peach Fruit Development

Apple has one *SHATTERPROOF* (*SHP*), two *AG*, and two *SEEDSTICK* (*STK*) homologs (Table 1; Figure 1A). When two *AG* homologs (*MdMADS15* and MdMADS22) are knocked out together the transgenic plants produce flowers with an increased number of petals, but these flowers still have stamens and carpels and can be pollinated to produce normal sized fruit with increased number of locules (Figure 3; Klocko et al., 2016). However, the simultaneous knockout of three genes, *MdMADS15/22*and *MdMADS14* (a *SHP* homolog), results in the flowers of these transgenic plants having increased numbers of petals and no stamens or carpels. This phenotype resembles the floral phenotype of Arabidopsis *agamous* mutants. The absence of the carpel in these apple mutant flowers means that they cannot be pollinated; however, they can be induced to produce pseudo fruit without any seed or fruit core by treatments with a combination of plant growth regulators including auxin, cytokinin, and gibberellin (Schaffer et al., 2017; Ireland et al., 2021). This is the first study to show pseudo fruit development without the development of carpel tissue. It is not fruit development in the true sense but extra-carpellary tissue continuing its development when carpels are not formed.

In peach, expression of *PrpPLENA*, a homolog of *SHP*, increases during fruit ripening. Ectopic expression of *PrpPLENA* in tomato plants results in the conversion of sepals into carpel-like structures that, like real fruits, become fleshy and ripen. These transgenic tomatoes also show accelerated ripening (Tadiello et al., 2009). This phenotype is the same as that of tomato over-expressing a *SHP* homolog *TAGL1* (Vrebalov et al., 2009), indicating that *PrpPLENA* is likely the functional homologue of tomato’s *TAGL1*. Another class C-related gene of peach, *PrpSHP*, is expressed from the full anthesis until the fruit harvest stages. *PrpSHP* expression levels differ between those peach cultivars sensitive to split-pit and those resistant to split-pit (Tani et al., 2007). These findings suggest that temporal regulation of *PrpSHP* expression may influence the split-pit process of peach, which is a process similar to the silique dehiscence in Arabidopsis regulated by *SHP1* and *SHP2* (Liljegren et al., 2000).

The *AG/SHP* homologs of apple and peach have similar functions in regulating the growth of carpellary tissues during fruit development. The difference is that the carpellary tissues contribute to the growth of the whole fruit in peach, but only to the fruit core of apple. As demonstrated in the MADS15/15/22 knockout transgenic plants coreless apple pseudo fruit can be induced to develop from the extra-carpellary hypanthium when the function of all *AG/SHP* homologs is lost. In contrast, complete loss of function of *AG/SHP* homologs in peach would likely result in no fruit being produced, although such experiments have yet to be reported.

## *Sep*-Related Genes Regulating Fruit Flesh Growth And Ripening

The class E *SEP* genes show partial redundancy in *Arabidopsis* with single *SEP* mutants showing only subtle phenotypes, in contrast to the triple *sep1/2/3* mutant which displays indeterminate flowers and whorls of sepals (Pelaz et al., 2000) and the quadruple *sep1/2/3/4* mutant which has whorls of leaf-like organs but no flower-like structures (Ditta et al., 2004). The role of *AtSEP* genes as floral meristem identity genes has been highlighted by their over-expression. Plants with a *SEP4* transgene under the control of the CaMV35S promoter exhibit replacement of the inflorescence meristem with fused terminal flowers (Ditta et al., 2004), while plants with a *SEP3* transgene expressed from the CaMV35S promoter are early flowering with solitary flowers subtended by curled cauline leaves (Pelaz et al., 2001).

The functions of *SEP* homologs in regulating fruit development have been shown in tomato. Suppression of expression of either the *AtSEP1/2*-like gene *TM29* or *AtSEP3*-like gene *TM5* promotes parthenocarpic fruit development in transgenic tomato (Pnueli et al., 1994; Ampomah-Dwamena et al., 2002). This suggests that *SEP* genes repress tomato fruit development. Vrebalov et al. (2002) demonstrated that the *AtSEP4*-like *LeMADS-RIN* (*RIN*) gene is a “master regulator” of fruit ripening in tomato. *RIN* is proposed to act upstream of, and independently to, ethylene-mediated regulation of ripening. *Rin* mutant plants show inhibition of all ripening-related traits, including autocatalytic ethylene production, carotenoid accumulation, softening, and volatile production. The *rin* mutant fails to ripen even under ethylene treatment (Vrebalov et al., 2002). Furthermore, RIN can bind and activate the promoter of ethylene responsive genes, such as *LeACS2* (Ito et al., 2008) and in the *rin* mutant, the expression of *LeACS2* is suppressed (Barry et al., 2000).

In strawberry (*Fragaria* x *ananassa* Duch.) downregulation of *FaMADS9* (an *AtSEP1/2*-like gene) results in increased green coloration of petals toward a sepaloid form (Seymour et al., 2011), similar to the sepaloid petals seen in *Arabidopsis sep3* single mutants (Pelaz et al., 2001). Seymour et al. (2011) also showed that although fruit development appears normal when *FaMADS9* is downregulated, ripening is delayed with respect to the accumulation of anthocyanin, degreening of achenes, and fruit softening, akin to the *SEP4*-like *rin* mutant of tomato. Furthermore, complete suppression of *FaMADS9* leads to severe inhibition of fruit development such that the receptacle develops only to an immature stage (Seymour et al., 2011). Another study showed that over-expression of *FaMADS1α*, also belonging to the *SEP1/2* clade, reduced the expression of anthocyanin-related genes and delayed fruit ripening (Lu et al., 2018). Point mutations of *FveSEP3* (FvH4_4g23530) generated by EMS treatment and CRISPR/Cas9-mediated gene editing convert petals, stamens, and carpels to sepaloid organs, promote parthenocarpic fruit growth, and delay fruit ripening in woodland strawberry (Pi et al., 2021).

In apple (*Malus* x *domestica*), suppression of *MdMADS8/9*, a member of the *SEP1/2* clade, partially converts petals to sepals and reduces fruit flesh growth, resulting in small fruit (Figure 3). At fruit maturity, the *MADS8/9*-suppressed apples produce no ethylene and fail to ripen, showing no starch degradation or ethylene-modulated ripening traits (Ireland et al., 2013). Unlike the tomato *rin* mutant, the *MADS8/9*-suppressed apples ripen when supplied exogenous ethylene, suggesting that in this case the *SEP*-like genes are not acting as a master regulator controlling the competency of the fruit to ripen, but instead control the developmental regulation of distinct facets of apple fruit ripening, including the initiation of ethylene production.

Over-expression of the peach (*Prunus persica*) *PrpMADS5*, an *AtSEP3*-like gene, in *Arabidopsis* results in an early flowering phenotype and over-expression of the peach *PrpMADS7, an AtSEP1/2*-like gene causes extremely early flowering, with bolting after the cotyledon stage (Xu et al., 2008). The *PrpSEP1* gene is closely related to apple *MdMADS8 and MdMADS9* and is expressed in peach flesh. Its expression pattern during the storage of melting flesh peach is consistent with that of ethylene- and ripening-related genes. Suppression of *PrpSEP1* expression by virus-induced gene silencing (VIGS) delays fruit ripening and softening in melting flesh peach. PrpSEP1 can interact with the promoter of a ripening-related gene encoding a cell wall softening enzyme polygalacturonase (PG; Li et al., 2017).

From the studies discussed above, it is clear that *SEP* homologs play similar function in regulating fruit ripening among apple, peach, and strawberry. When the expression level of *SEP* homologs is reduced, fruit ripening is delayed in all three species. However, *SEP* homologs show opposite functions in regulating fruit flesh growth between apple and strawberry, i.e., promoting flesh tissue growth in apple (Ireland et al., 2013) but inhibiting flesh tissue growth function in strawberry (Pi et al., 2021). This difference may be related the difference of floral organs contributing to development of fruit flesh between apple and strawberry. Apple fruit flesh develops from a hypanthium containing the bases of sepals, petals, and stamens, whereas strawberry flesh develops from a receptacle known as a stem tip. However, further research is required to understand the mechanisms of this differential regulation.

## Conclusion and Future Prospects

**Figure 5:**
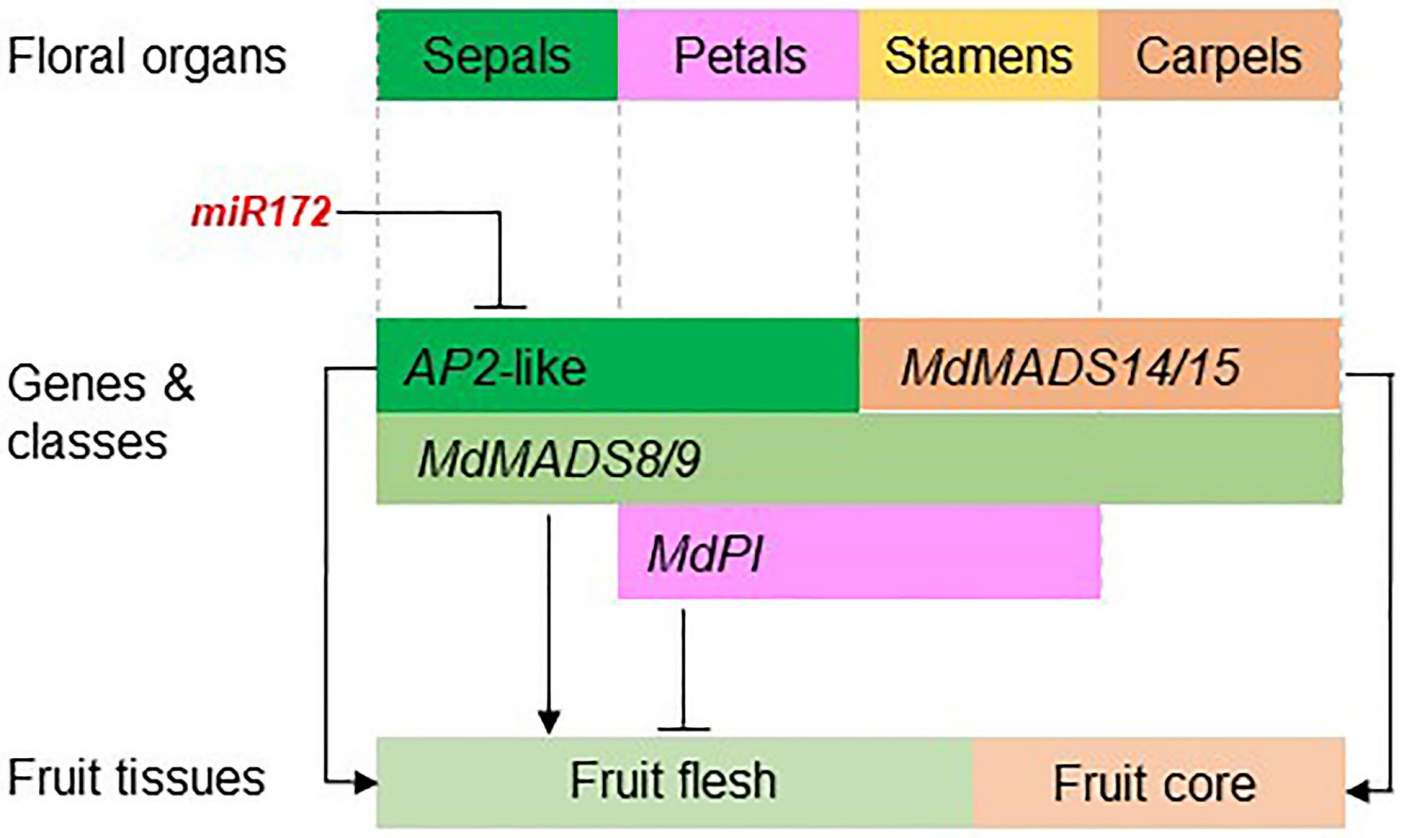
The apple floral organ genes regulate fruit development. The diagram shows the apple floral organ genes that have been functionally studied in apple transgenic plants. miR172 inhibits the expression of *AP2-like* genes that promote apple fruit flesh growth (Yao et al., 2015). Ectopic expression of *MdPI* inhibits fruit flesh growth (Yao et al., 2018) while knockout of *MdPI* promotes parthenocarpic fruit development (Yao et al., 2001). The *AG/SHP* homologs *MADS14/15* are required for fruit core growth and coreless fruit can be produced by knocking out *MADS14/15* (Schaffer et al., 2017; Ireland et al., 2021). The class E genes *MdMADS8/9* are required for fruit flesh growth and fruit ripening (Ireland et al., 2013).

The studies on apple floral MADS-box genes started in the mid-1990s. Recent advancements of reference genome assembly for multiple Rosaceae species have facilitated the genome-wide identification of floral MADS-box and *AP2* genes, and their expression patterns in developing fruit tissue have been analyzed using transcriptome data. These genome and transcriptome resources have allowed identification of promising candidate genes for analyzing their function in the regulation of fruit development and quality. So far, functional analyses of these genes have been predominantly in apple and strawberry because these two species are relatively easy to transform, and strawberry has a relatively short generation time. The function of a few genes, such as *MdPI* and miR172, has also been shown using natural mutants. The functional analyses of floral organ genes in regulating apple fruit development are summarized in Figure 5.

Future studies should be directed to functionally analyzing the roles of floral organ genes in a wider range of Rosaceae species for a greater understanding of the molecular mechanisms regulating fruit type diversity and fruit development in the family. Gene editing, using CRISPR-Cas9 or similar, offers the opportunity to disrupt the function of specific gene family members to elucidate their precise role, the degree of redundancy within the gene family and any neo- and sub-functionalization that has evolved. However, as most Rosaceae plants are woody perennials, the challenges of elucidating a gene’s role in the regulation of fruit development and fruit quality are the relatively long time to the plants first flowering and fruiting and their recalcitrance to transformation. These challenges may be overcome by using transgenes to promote early flowering, as has been achieved using the *Betula pendula* (silver birch) *BpMADS4* gene to accelerate apple flowering (Weigl et al., 2015), and transgenes to enhance the efficiency of plant transformation and regeneration, as has been achieved using the *Baby boom* gene to improve maize and apple transformation (Lowe et al., 2016; Chen et al., 2021). In addition, recent accumulation of genome sequence data from a large number of cultivars and germplasm accessions of some of the Rosaceae family, along with transcriptomics data, is proving useful for the identification of alleles associated with key fruit development traits. With the ongoing expansion of genomics data, more refined spatio-temporal transcriptomics, new gene editing, and transformation technologies and their application across a wider range of Rosaceae family members, we would expect that significantly faster progress will be made in the next decade to understand how floral *MADS-box* and *AP2* genes regulate fruit development and fruit quality in Rosaceae, a family that produces such a wide array of fruit types.

## Author Contributions

All authors contributed to the design of the review and approved the manuscript. J-LY completed the first draft. J-LY, CK, and AG revised the manuscript.

## Conflict of Interest

Authors AG and J-LY were employed by company The New Zealand Institute for Plant and Food Research Limited.

The remaining authors declare that the research was conducted in the absence of any commercial or financial relationships that could be construed as a potential conflict of interest.

## Publisher’s Note

All claims expressed in this article are solely those of the authors and do not necessarily represent those of their affiliated organizations, or those of the publisher, the editors and the reviewers. Any product that may be evaluated in this article, or claim that may be made by its manufacturer, is not guaranteed or endorsed by the publisher.
